# Medical image fusion quality assessment based on conditional generative adversarial network

**DOI:** 10.3389/fnins.2022.986153

**Published:** 2022-08-09

**Authors:** Lu Tang, Yu Hui, Hang Yang, Yinghong Zhao, Chuangeng Tian

**Affiliations:** ^1^School of Medical Imaging, Xuzhou Medical University, Xuzhou, China; ^2^School of Information and Electrical Engineering, Xuzhou University of Technology, Xuzhou, China

**Keywords:** attention mechanism, conditional, generative adversarial networks, image quality assessment, medical image fusion

## Abstract

Multimodal medical image fusion (MMIF) has been proven to effectively improve the efficiency of disease diagnosis and treatment. However, few works have explored dedicated evaluation methods for MMIF. This paper proposes a novel quality assessment method for MMIF based on the conditional generative adversarial networks. First, with the mean opinion scores (MOS) as the guiding condition, the feature information of the two source images is extracted separately through the dual channel encoder-decoder. The features of different levels in the encoder-decoder are hierarchically input into the self-attention feature block, which is a fusion strategy for self-identifying favorable features. Then, the discriminator is used to improve the fusion objective of the generator. Finally, we calculate the structural similarity index between the *fake* image and the *true* image, and the MOS corresponding to the maximum result will be used as the final assessment result of the fused image quality. Based on the established MMIF database, the proposed method achieves the state-of-the-art performance among the comparison methods, with excellent agreement with subjective evaluations, indicating that the method is effective in the quality assessment of medical fusion images.

## Introduction

As the population aging becomes familiar, and the vulnerability of the human brain to physical, chemical, and viral attacks, the incidence of brain diseases such as intracranial tumors, intracranial infectious diseases, and cerebrovascular diseases is gradually increasing, which has seriously threatened human health and wellbeing (Chen et al., 2022; Gottesman and Seshadri, 2022). There are many medical imaging modalities for clinical diagnosis and treatment of brain diseases, including computed tomography (CT), magnetic resonance imaging (MRI), positron emission tomography (PET), and so on. Different imaging methods always have their unique advantages in attracting clinicians to choose (Liu et al., 2019; Cauley et al., 2021; Preethi and Aishwarya, 2021). For example, CT could superbly display the histological structure of the skull and the density changes in the brain parenchyma, while MRI could faithfully restore the essential features of the nervous or soft tissue. Generally, it is difficult for medical experts to identify the necessary information from a single modality of brain images to ensure the reliability of clinical diagnosis (Townsend, 2008). Additionally, some early work found that radiologists could effectively improve the diagnostic accuracy if they can analyze imaging results of more than two modalities at the same time (Li and Zhu, 2020). From a technical point of view, multimodal medical image fusion (MMIF) just meets this clinical need. Therefore, recently, MMIF has received attention and extensive exploration by researchers (Li et al., 2020; Ma et al., 2020; Liu et al., 2021).

The purpose of MMIF is to complement the image in different modalities to obtain better image expression, quality, and information perception experience (Azam et al., 2022; Liu et al., 2022). The fused images may contain both anatomical structure and tissue metabolism information (e.g., image fusion of CT and MRI), which improves the applicability of image-based diagnosis or assessment of diseases, thereby simplifying diagnosis. At present, many high-quality MMIF methods have been proposed (Arif and Wang, 2020; Wang K. P. et al., 2020; Duan et al., 2021; Ma et al., 2022; Xu et al., 2022). Madanala and Rani (2016) proposed a two-stage fusion framework based on the cascade of discrete wavelet transform (DWT) and non-subsampled contour transform (NSCT) domains, realizing the combination of spatial domain and transform domain. Inspired by the Tchebichef moments’ ability to effectively capture edge features, Tang et al. (2017) used the Tchebichef moments energy to characterize the image shape, and thus designed an MMIF method based on the pulse coupled neural network (PCNN). However, the performance evaluation of these MMIF models and fused images has not been fully explored.

Normally, the higher the image quality, the more features and information human observers can receive or perceive through the image. As the ultimate observers and beneficiaries of the fused images, medical experts, although they subjectively evaluate the fused images as the most direct and reliable solution, it will be a very time-consuming and labor-intensive task, and it is not very useful in practical applications. Hence, objective image quality assessment (IQA) is very necessary (Liu et al., 2018; Shen et al., 2020; Wang W. C. et al., 2020). Some existing objective quality assessment studies include deblocking images, screen content images, multiple distorted images, and noisy images, etc. (Gao et al., 2008; Min et al., 2019; Liu et al., 2020; Meng et al., 2020). For instance, in early work, Wang et al. (2004) developed structural similarity (SSIM) index based on the subjective perception of image structure information, which achieved a breakthrough in the objective evaluation of image quality. Kang et al. (2014) used deep learning techniques to accurately predict the quality of images without reference images, and their method greatly improved the performance and robustness of the algorithm. On the premise of highlighting the important detection objects, Lei et al. (2022) fuses multiple features of the images at the pixel level and designed an IQA method of main target region extraction and multi-feature fusion. However, among these IQA methods, they are proposed for general use in the field of image fusion, not specifically for MMIF. Note that the quality assessment of medical fusion images includes information fidelity, contrast, grayscale tolerance, and region of interest (ROI). In clinical practice, the ROI usually refers to the lesion area. And, the ROI has a great influence on the results of IQA, which is the most different from the natural image (Du et al., 2016a; Cai et al., 2020; Chabert et al., 2021). As a result, there is an urgent need for a dedicated objective IQA method for medical fusion images.

We discussed with radiologists and found that the quality of a medical fusion image mainly depends on its impact on disease diagnosis. That is, the medical fusion image retains disease-relevant information in the ROI, it will be acceptable and will be given a higher subjective evaluation score. To this end, we propose a novel medical fusion image quality assessment method that uses the radiologist’s mean opinion scores (MOS) as the constraint on conditional generative adversarial networks (GANs). Concretely, the method firstly extracts the feature of different depths from MOS and two input source images with the aid of dual-channel encoder-decoder. Next, under the supervision of the attention mechanism, we fuse the feature information hierarchically, and generate the fused image through the up-sampling algorithm. Then, the discriminator (***D***) differentiates the source of the fused images to improve the generator (***G***) performance. Finally, we calculate the SSIM of the *fake* image and *true* image, and the constrain value corresponding to the maximum value of SSIM as the evaluation result. The experimental results show that the proposed method is superior to the previous IQA algorithms, and the objective results obtained are more consistent with the subjective evaluation of radiologists.

The content of this paper is arranged as follows. In see section “Methodology,” the proposed method is mainly introduced from four aspects: Encoder-Decoder, ***G***, ***D***, and objective function. The details of the experiments are presented in see section “Experiments.” See section “Discussion and conclusion” contains the discussion and conclusion of this paper.

## Methodology

**FIGURE 1:**
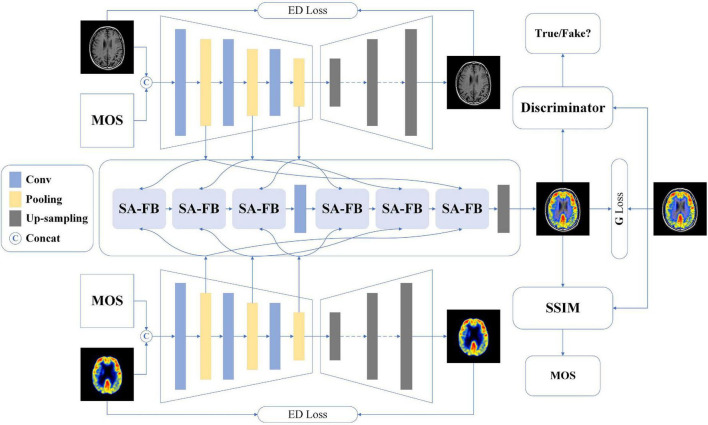
The overall architecture of our proposed method.

The structure of our proposed model based on conditional generative adversarial network is shown in Figure 1, and the details are described below.

### Dual-channel encoder-decoder

Among the existing multimodal medical images, each image has its unique imaging method and the advantage of displaying different human tissue. Therefore, accurately extracting the latent and deep key features of each modality image will be extremely conducive the image fusion (Ma et al., 2019). Besides, we also hope that MOS, the gold standard for image quality assessment, can participate in the feature extraction process of model learning images, in other words, learning the non-linear mapping relationship between MOS and fused images. To achieve this vision, we develop a dual-channel encoder-decoder structure.

First of all, we encapsulate three convolutional blocks, each of which contains two sets of convolutional layers, batch normalization (BN) layers, and activation layers. Specifically, the filter, stride, and padding of each convolutional layer are 3 × 3, 1, and 1, respectively. BN operation can effectively accelerate the network training as well as alleviate the problem of over-fitting. Thus, we append such operation after each convolutional layer. Considering that the image encoding process is important to learn image features and image fusion, we use a more comprehensive activation algorithm: Lleaky Rectified Line Unit (LeakyReLU). Then, we added max pooling operation instead of average pooling operation after each convolutional block. The reason is that the model should perform some specific feature selection under the constraints of MOS to learn more recognizable features. Each feature map output through the pooling operation is fed to the self-attention fusion block (SA-FB) separately, and more details will be explained in the next section. For the decoder, seven groups of deconvolution layer, BN layer, and Rectified Line Unit (ReLU) activation function layer complete the up-sampling operation of the feature maps. Finally, a reconstructed image of size 128 × 128 is obtained. It is worth noting that during the decoding operation, there is no feature map as output.

Perform the concatenating operation on the image of two different modalities (*MI*_*i*_,*i* = 1,2) and the corresponding MOS of their fused image, and the result is named *MI*_*imos*_, and then input into two encoder-decoders, respectively. The feature map after the pooling layer is represented as *F*_*ij*_, then the *j*-th feature map for the *i*-th modality can be marked as:


(1) $$F_{i\,j}=C\,o\,n\,v\,B\,{\left(M\,I_{i\,m\,o\,s}\right)}_{j}$$

where *ConvB*(•) means the operation process of the *j*-th convolution block. The integer value range of *j* is one to three as only three convolution blocks are established in the encoding process. Here, sum of absolute difference is employed as the loss function for single modality image restoration, as defined by the following equation:


(2) $$L_{E\,D}=\sum_{i}\sum \left|M\,I_{i}-\hat{M\,I_{i}}\right|,i=1,2$$

Where ${\hat{M\,I}}_{i}$ refers to the original modal image restored by the decoder, and *i* represent the two modal images input to the dual-channel encoder-decoder, respectively.

### Generator architecture

**FIGURE 2:**
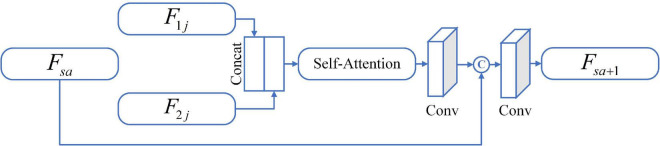
The diagrammatic sketch of SA-FB.

It is generally known that image fusion is the operation of synthesizing two or more images into one image, preserving the most representative features of each modality. To avoid the impact on image feature learning, independent of the dual-channel encoder-decoders, we design a feature fusion method based on the self-attention (SA) mechanism, as shown in Figure 1. Different levels of features contain different image information, for example, shallow features mean contour information while deep features represent texture information. For the three-level of feature *F*_*ij*_ yielded in the encoder, we develop the SA-FB to complete the fusion hierarchically. The structure diagram of SA-FB is shown in Figure 2.

In particular, the first SA-FB has only two inputs (i.e., *F*_*ij*_), and the fusion feature *F*_*sa*_ is null. We do not carry out any feature selection operations (such as taking extreme values) during inputting, but directly feed the initial features *F*_1*j*_ and *F*_2*j*_ to SA after concatenating, and SA will sign weights to the features. Such setting can replace the manual feature selection algorithm, thus avoiding the loss of important information. SA is a variant of the attention mechanism from Sergey and Nikos (2017). It could coarsely estimate the foreground region to find prominent features that are in favor of later search. At the same time, it also reduces the dependence on external information, and is better at capturing the internal relevance of features. Immediately after, we adopt a convolution layer at the end of the SA. The convolution kernel size is set to 1 × 1 with stride 1 for adapt the output feature map weights. The output of this convolutional layer is concatenated with *F*_*sa*_, and further input to a new convolution layer with a filter size of 3 × 3, and stride 1. In the end, a feature output *F*_*sa* + 1_ that has undergone a complete SA-FB is obtained, and can be expressed as:


(3) $$F_{s\,a+1}=safb\left(F_{i\,j},F_{s\,a}\right),\left(i=1,2,j=1,2,3\right)$$

where *safb*(•) is a series of operations of SA-FB. It should be mentioned that each convolution layer in the first three SA-FB is followed by BN layer and LeakyReLU as an activation function, which is similar to the encoder. The max pooling operation also appends after each SA-FB. The SA-FB in the up-sampling stage eliminates the pooling operation and changes the activation function to ReLU. On the basis of MOS as the condition to extract two modal image features, the ***G*** generates a fused image with 128 × 128. The parameters of the ***G*** are only renewed by the following loss function:


(4) $$L_{f\,u\,s\,i\,o\,n}=\frac{1}{N}\,\sum_{n=1}^{N}{\left|y_{t\,r\,u\,e}-\hat{y}\right|}_{1}$$

where *y*_*true*_ means the fused image with the corresponding MOS and the $\hat{y}$ represents the fused image produced by the ***G***. *N* is the total number of generations, and *n* represents the *n*-th generation. When training ***G***, minimize the following objective function:


(5) $$\begin{array}{c} L_{G}=V_{G}^{m\,o\,s}\,\left(G,D\right)=E_{M\,I_{1},M\,I_{2}∼P_{\text{data}\,M}}\\ \left[\log\left(1-D\,\left(M\,I_{1},M\,I_{2},\left(G\,\left(M\,I_{1},M\,I_{2}|m\,o\,s\right)\right)\right)\right)\right]+\alpha \,L_{f\,u\,s\,i\,o\,n} \end{array}$$

where *P*_*dataM*_ represents the distribution of *MI*_1_ and *MI*_2_, respectively, and *E*_*MI*_1_,*MI*_2_∼*PdataM*_ represents the expectation of *G*(*MI*_1_,*MI*_2_|*mos*). α is a weight hyperparameter and is set to 100 during training.

To sum up, we restrict the generator based on MOS conditional information, and achieve the goal of generating image content. This is similar to that the generator analyzes the fused image by simulating the human visual system (HVS) and learns the non-linear mapping relationship between MOS and image. That is, the generator simulates a radiologist to assess the quality of the fused image, there by producing a fused image that matches the quality of MOS (i.e., ***G*** has learned the evaluation experience of radiologist).

To evaluate the quality of the fused image *FI*_12_, first of all its original two modal images *FI*_1_ and *FI*_2_ should be input and then generate the fusion image *FI*_*fake*_ by ***G***. Where *1* and *2* represent two modal images, respectively. We have created five *fake* MOS (*MOS*_*k*_ = 0.2*k*,*k* ∈ [1,5],*k* ∈ ℤ) as the conditional constraints ***G***, so the *FI*_*fake*_ can be renewed to *FI*_*fake*,*k*_, which represents the fused image generated under the five constraints. Finally, the SSIM between *FI*_12_ and *FI*_*fake*,*k*_ is calculated, and the MOS corresponding to the optimal value is taken as the assessment result, as follows:


(6) $$Q=\max S\,S\,I\,M\,\left(F\,I_{12},G\,\left(F\,I_{1},F\,I_{2}|M\,O\,S_{k}\right)\right)$$

### Discriminator architecture

The discriminator needs to determine whether the generated image conforms to the real data distribution, so its structure is much simpler than the generator. In the proposed method, the input of the ***D*** is the generated fusion image or the original fusion image, all of which are 128 × 128 in size, and down-sampling is implemented using the discriminator block (DB). Each DB consists of a convolution layer with a filter size of 3 × 3, stride of 2 and padding of 1, and followed by BN processing. The LeakyReLU is used as the activation function for each block. The image passes through four DB in sequence, and after each DB, the size of the feature map becomes a quarter of that before input. An independent convolutional layer with convolution kernel 3 × 3 and stride 1 is appended to the last DB, and the final obtained feature map is 6 × 6. At last, the discriminator will judge the authenticity of the result. We apply mean square error (MSE) as the loss function to optimizing the parameters of the ***D***. Further, the objective function of ***D*** can be reformulated as:


(7) $$\begin{array}{c} L_{D}=V_{D}^{m\,o\,s}\left(G,D\right)=E_{y_{\text{true}}∼P_{d\,a\,t\,a}}[\log D(y_{t\,r\,u\,e}\\ \left|mos\right)]E{}_{M\,I_{1},M\,I_{2}∼P_{d\,a\,t\,a\,M}}[\log\left(1-D\left(G\left(MI_{1},MI_{2}|mos\right)\right)\right)] \end{array}$$

where *P*_*data*_ represents the distribution of *y*_*true*_ and *E*_*ytrue*_∼*P*_*data*_ represents the expectation of *y*_*true*_.

### Total objective loss function

As shown in Figure 1, we use MOS as a condition to limit the content of the image generated by ***G***, and ***D*** determines whether the distribution of the generated fused image is true or false. ***G*** and ***D*** are trained against each other, and finally achieve the goal of Nash Equilibrium. Therefore, the optimization process of the whole network can be expressed by Eq. 8:


(8) $$L_{a\,l\,l}=\min_{G}\max_{D}V\,\left(G,D\right)+\beta \,L_{E\,D}$$

where *V*(*G*,*D*) can be obtained by Eqs. 5 and 7, respectively. β is a weight hyperparameter and is set to 20 in this experiment.

## Experiments

### Dataset

**FIGURE 3:**
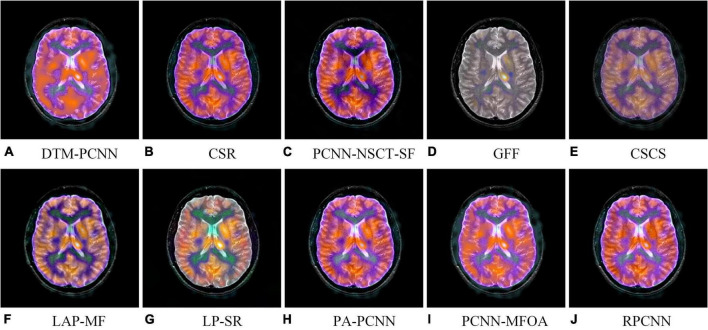
An example of fused images generated by ten different MMIF algorithms. Algorithms include **(A)** discrete Tchebichef moments and pulse coupled neural network (DTM-PCNN) (Min et al., 2019), **(B)** convolutional sparse representation (CSR) (Liu et al., 2016), **(C)** pulse-coupled neural network with modified spatial frequency based on non-subsampled contourlet transform (PCNN-NSCT-SF) (Das and Kundu, 2012), **(D)** guided filtering (GFF) (Li et al., 2013), **(E)** cross-scale coefficient selection (CSCS) (Shen et al., 2013), **(F)** union Laplacian pyramid with multiple features (LAP-MF) (Du et al., 2016b), **(G)** Laplacian pyramid and sparse representation (LP-SR) (Liu et al., 2015), **(H)** parameter-adaptive pulse-coupled neural network (PA-PCNN) (Yin et al., 2019), **(I)** pulse coupled neural network using the multi-swarm fruit fly optimization algorithm (PCNN-MFOA) (Tang et al., 2019), and **(J)** reduced pulse-coupled neural network (RPCNN) (Das and Kundu, 2013).

Image quality assessment has been developed in full swing in many fields and has made substantial progress. But, in the past period, the short-lived time of the MMIF algorithm has resulted in few research dedicated to the quality assessment of medical fusion images. In order to enable the medical image fusion algorithm to restore the brain structure more accurately and reflect tissue metabolic information more objectively, meeting the needs of clinical diagnosis, based on our previous work (Tang et al., 2020), we construct a special multimodal medical image fusion image database (MMIFID) with subjective evaluation of radiologists. Particularly, this work uses brain images from the AANLIB dataset, provided by Harvard Medical School and accessible online. The image size is 256 × 256, which can be browsed directly on the online web page. Most importantly, since image registration is completed for each combination of different modal images, it is one of the most widely used datasets. We selected 120 pairs of images in the AANLIB dataset and fused the images through ten image fusion algorithms. Figure 3 shows examples of results generated by ten fusion algorithms. Consistent with our previous work (Tang et al., 2020), radiologists subjectively evaluated the quality of the fused image and gave a score (1 is the lowest and 5 is the highest), and finally obtained the MOS.

### Evaluation metrics

To comprehensively evaluate the performance of the proposed method, that is, the consistency of the model’s assessment of the fused image quality with the MOS score, we adopted four commonly used performance metrics: Spearman Rank-order Correlation Coefficient (SRCC), Kendall Rank-order Correlation Coefficient (KRCC), Pearson Linear Correlation Coefficient (PLCC), and Root Mean Square Error (RMSE). To sum up, the higher SRCC, KRCC and PLCC value and lower RMES value mean better model performance. Note, the model is evaluated at the end of each training epoch, and the final model is the checkpoint model with the best evaluation performance within 200 epochs.

### Comparison methods

The results are compared with those of the state-of-the-art (SOTA) image fusion quality metrics, which are listed as follows:

Mutual Information (*Q*_*MI*_) (Hossny et al., 2008): As an objective method for evaluating image fusion performance, this method can measure the features and visual information from the input initial image and the fused image. The MI method we adopted is optimized by Hossny et al. (2008).

Non-linear Correlation Information Entropy (*Q*_*NCIE*_) (Wang and Shen, 2004): Wang et al. propose a method based on non-linear correlation measures. This method evaluates the performance of image fusion algorithms by analyzing the general relationship between the source image and the fused image.

Gradient based fusion metric (*Q*_*G*_) (Xydeas and Petrovic, 2000): This performance metric measures the amount of visual information transmitted from the source image to the fused image.

Ratio of spatial frequency error (*Q*_r*SFe*_) (Zheng et al., 2007): This is a new metric based on extended spatial frequencies, and its original intention is to guide the algorithm to obtain a better fusion image.

The metric proposed by Yang et al. (2008) (*Q*_*Y*_): According to the structural similarity between the source image and the fused image, this method treats redundant regions and complementary/conflicting regions, respectively.

A metrics based on edge preservation (*Q*_*EP*_) (Wang and Liu, 2008): An image fusion metric method is proposed based on the perspective of edge information preservation.

A metric based on an absolute image feature measurement (*Q*_*P*_) (Zhao et al., 2007): Based on phase congruency and its moments, a pixel-level image fusion performance metric is defined, which provides an absolute measure of image features.

Table 1 shows the performance of the above methods on our MMIFID, and the last row is the performance of the method proposed in this paper. Generally, SRCC, KRCC, and PLCC can measure the agreement between MOS and the objective scores, while RMSE can calculate its absolute error. Thus, the higher the SRCC, KRCC, and PLCC values, the better the quality evaluation metrics. The smaller the RMSE, the higher accuracy of the assessment. From Table 1, we can observe that the proposed method outperforms all SOAT methods. Furthermore, it can also be noticed that our proposed metrics are obviously better than these methods, which especially highlights that the quality assessment methods for medical images differ from natural images. Therefore, it is necessary to explore the special indicators for the quality evaluation of medical fusion images.

**TABLE 1:** Comparison of quality assessment performance of different models.

Methods	SRCC	KRCC	PLCC	RMSE
*Q*_*MI*_	0.2545	0.3604	0.2772	0.3804
*Q*_*NCIE*_	0.2647	0.3608	0.2920	0.4093
*Q*_*G*_	0.2488	0.3322	0.2444	0.2791
*Q*_r*SFe*_	0.1801	0.2076	0.3126	0.2872
*Q*_*Y*_	0.1884	0.2400	0.2503	0.4002
*Q*_*EP*_	0.0960	0.1275	0.2235	0.2970
*Q*_*P*_	0.1093	0.1216	0.0803	0.3007
Proposed	**0.8259**	**0.7426**	**0.8197**	**0.1709**

The bold values are the results of our proposed method, which achieves the best performance.

### Ablation experiment

As we know, image fusion can be divided into two categories: early fusion and late fusion. The early fusion fuses the image directly together and then carries on the process of feature extraction and selection, while the late fusion allows the images to go through the process of feature extraction and selection, respectively, and then perform image feature fusion. Therefore, our two ablation experiments are to downgrade the proposed method to the early fusion and late fusion model, named Early-FM and Late-FM, respectively. Specifically, Early-FM first concatenates *F*_1_, *F*_2_ and MOS, and then completes feature learning through the single-channel encoder-decoder structure (e.g., we use the single-channel encoder-decoder to replace dual-channel encoder-decoder). The features output by the third convolutional block will be used to generate the fused image. Different from Early-FM, the Late-FM first concatenates the images of the two modalities and their respective MOS, and then inputs them to the dual-channel encoder-decoder, respectively, to complete feature learning. The third convolution block of the two channels outputs features, and the fused features are obtained by fusion operation. Finally, ***G*** generates the fused image. For the third ablation experiment, we eliminated the SA mechanism in SA-FB, and the rest of the structure is consistent with the proposed method, which is marked as proposed w/o SA. We train the Early-FM, Late-FM and the proposed w/o SA based on the same method applied in the proposed method and tabulate their test performances in Table 2.

**TABLE 2:** Comparative results of ablation experiments.

Methods	SRCC	KRCC	PLCC	RMSE
Early-FM	0.7077	0.6208	0.6779	0.2425
Late-FM	0.7288	0.6427	0.6833	0.2417
Proposed w/o SA	0.7825	0.7113	0.7867	0.2020
Proposed w SA	**0.8259**	**0.7426**	**0.8197**	**0.1709**

The bold values are the results of our proposed method, which achieves the best performance.

Two main conclusions can be drawn from the experimental results. First, the performance results of both Early-FM and Late-FM are worse than those of the hierarchical fusion strategy we designed (i.e., the proposed method without or with SA). More concretely, the results comparison between Early-FM and proposed method are notably improved by 11.82% for SRCC, 12.18% for KRCC, and 14.18% for PLCC, while the RMSE decreased by 7.16%. For Late-FM, the proposed method also improves SRCC, KRCC, and PLCC by 9.71, 9.99, and 13.64%, respectively, while reducing RMSE by 7.08%. It is conceivable that the unnecessary noise in the early fusion will affect the quality of the fused image, and the late fusion may lose important details of the image. Thus, the obtained results are not pleasing. Second, the performance of the proposed method with SA as guidance is better than that without SA, which means that with the assistance of the SA mechanism, the process of model learning features is superior.

## Discussion and conclusion

Multimodal medical image fusion, as a way to express multimodal diagnostic information at the same time, has gradually gained attention in the field of medical imaging. However, the diagnostic information that a radiologist can perceive is *not only* related to the amount of initial image information contained in the fused image, *but also* to the quality of the fused image. Therefore, the quality assessment of MMIF plays an increasingly important role in the field of image processing and medical imaging diagnosis. At the same time, it has also aroused the interest of many scholars in the industry.

As MMIF is gradually gaining recognition in the medical field, quality assessment of fused images has also developed vigorously as an emerging field. An excellent objective assessment method can *not only* achieve the purpose of image quality control, *but also* guide the optimization of image fusion algorithms, so as to find the best algorithm for image fusion of different modalities. For instance, a certain algorithm can achieve very good results for image of MRI and CT, but it is not suitable for image fusion of MRI and SPECT, and maybe another algorithm should be more suitable. Unfortunately, most of existing IQA research methods are based on natural images, and it is difficult to achieve satisfactory performance for medical fusion images (see section “Comparison methods”). On the basis of previous work, we augmented the medical image database, MMIFID, which takes the doctor’s MOS as the gold standard for subjective evaluation. The image content generated by ***G*** is constrained by MOS as a condition, and the non-linear mapping relationship between subjective evaluation and fused image is learned. The experimental results show that the objective evaluation results obtained from the model can match the subjective evaluation values well. In addition, compared with other IQA algorithms, we found that the proposed method outperforms the SOTA methods. Finally, we enumerate the potential limitations of this work as follows: (1) Although the database we built, as far as we know, is the largest multimodal medical image fusion database with MOS. However, it may still be a challenge for training GANs. In the future, we will continue to work on expanding the database. (2) Currently, the images contained in MMIFID are brain data, and we hope to add other body parts to the database in the future. (3) This work uses SSIM to calculate and obtain the final fusion image quality evaluation results, which may affect the accuracy of assessment to a certain extent. It would be better if the final evaluation result could also be directly assigned by GANs. Future, we will continue to explore the impact of fusing two modalities image through different methods, and design another novel IQA algorithm based on the idea of no reference.

## Data availability statement

The original contributions presented in this study are included in the article; further inquiries can be directed to the corresponding author. The brain images are accessible online: https://www.med.harvard.edu/aanlib/home.html.

## Author contributions

LT and HY wrote the main manuscript and contributed to the final version of the manuscript. CT and YH implemented the algorithm and conducted the experiments. YZ supervised the project and collected the data. All authors contributed to the article and approved the submitted version.
